# Dr. Rogers, on Mezereum

**Published:** 1801-03

**Authors:** J. Rogers

**Affiliations:** Great Russel Street, Bloomsbury


### *To the Editors of the Medical and Phyfical Journal.*

Gentlemen,

If the following few remarks on the external ufe of the inner bark of the Daphne Mezereum, may be thought worthy a place in the Medical and Phyfical Journal, I beg that you will infert them.

Linnæus claffes feveral fpecies of Daphne, but as I am acquainted with only two of them, the Daphne Mezereum, and Daphne Thymelæa, I fhall confine myfelf to thefe only.

When, about thirty years ago, I firft went to Ruffia, I heard of the bark of the Daphne Thymelæa being ufed as a fubftitute for iffues, and was advifed to try it.

Happy to hear that any method had been thought of to produce duce an uniform difcharge from the fkin, on any part of the body, fo as to obviate the neceffity of incifion, an operation often very much dreaded, efpecially by children, and always painful, I did not hefitate, the firft opportunity that offered, to give it a trial, which I did, by cutting a piece of the intire bark into the dimenfion of about three-quarters of an inch fquare, and fteeping it in vinegar fix hours; I then applied its inner furface to that part of the arm where iffues are generally cut, and faftened it on with a bandage.

After letting it remain on the arm 24 hours, I took it off, and there appeared on the part where it had been placed, a flight degree of inflammation, and the patient faid it itched a little. I then prepared another piece of bark in the fame manner as before, and laid it on as on the former day, and fo continued to do every day, till an excoriation of the fkin and fuppuration took place, which were kept up by the fame means for about a year, when the patient, a young fubject, who had been afflicted with a fcrophulous complaint of the eye-lids, got quite well. After leaving off the bark, a few gentle purges were ordered, and no ill confequence happened to the conftitution from ftopping the difcharge from the wound.

The Thymelæa, the fpecies of Daphne I made ufe of in the above cafe, was at that time exceedingly dear, and it was chance alone that made me acquainted with the Daphne Mezereum,* the fort that I have made ufe of ever fince, and which now occupies a place in the Pharmacopœa Roffica.

For the fpace of five and twenty years I have hardly ever ordered an iffue to be cut, but have trufted to the Mezereum bark alone, where a difcharge by the fkin has been thought neceffary; and have feen a great variety of herpetic and fcrophulous affections, where it has been employed with evident good effects. In ufing it, care fhould be taken to change it every morning, and to clean the fuppurating part, as is done in common iffues.

Sometimes the inflammation on the arm is confiderable, and the fuppuration abundant; yet neither of thefe fymptoms is ever attended with any ill confequence, their violence being foon abated by laying afide the bark for a few days, and wetting the part now and then with a little Aq. Saturnina; afterwards,

---

* An old Finnifh woman, who lived fervant in a houfe that I attended, feeing me ufe the bark, and hearing that it was very dear, told me, that a fhrub, with a bark very much like that which I employed, grew in great abundance in Finland, in the neighbourhood of Peterfburgh. I defired her to procure me fome, which fhe did, and on examination it proved to be the Daphne Mezereum, which, on trial, I found to have the fame effect as the other fort, except that it acted more fuddenly and vigoroufly on the fkin.

wards, its ufe may be renewed and continued as before. This method of promoting fuppuration has the advantage of the pea iffue, for the reafon above mentioned, in obviating terror, and it is not nearly fo painful as a perpetual blifter, at the fame time it is more under the controul of the furgeon.

After having faid thus much of the convenience of the above method of exciting inflammation and fuppuration, in preference to other means for the fame purpofe, it may not be improper to remark, that I have long indulged an idea that (although the abforption be but fmall from the bark) it may have a fpecific virtue towards curing many difeafes of the fkin. As I have had occafion to mention the Pharmacopœa Roffica, which, at leaft the laft edition, I prefume not to be in the hands of many medical gentlemen in this country, I fhall take the liberty to tranfcribe from it the whole of the article under the title Mezereum.

Mezerei cortex, radix. Daphne Mezereum, L. Cl. viij. Ord. Monogyn.

Frutex in Roffia feptentrionali, et Siberia fpontaneous.

Odor corticis ficci nullus.

Sapor fi diu mafticatur valde acris, fauces exurens, recentis acerrimus, inhærens.

Virtus in pauca dofi refolvens, in majori draftica, cardialgica, deurens.

Ufus. Exutorium fonticulorum, tumores fchirrofi et venerei ut tophi venerei, dolores nocturni, tumores tonfillarum, tefticulorum, colli, parotidum, ulcera venerea, acres humores alicubi ftagnantes.

Dofis. Radicis Mezerei drachmæ duæ, totidem liquiritiæ cum aquæ libris tribus ad libras duas decoctum, quod per diem confumitur; cataplafma ex decocto hocce, et farina; corticis ficci, aceto vini, vel aqua, per octo vel decem horas macerata ftruftrum, longitudinis pollicis, latitudinis fex vel octo linearum, imponitur plerumque brachio, fubter loco, cui inferitur mufculus deltoideus, vel aliis partibus, profciti medici indicatione et fpleniolo tectum fafcia firmatur.

Præter hunc fruticem aliæ Daphne funt fpecies, quæ corticem viribus priori fupporem erogant, ut Daphne Thymelæa, Daphne Gnidium, L. et hicce eft cortex verus (ecorce de Garou) Gallorum et Daphne Laureola, L.

Notwithftanding I have mentioned three-quarters of an inch fquare, and the Pharmacopœa Roffica has prefcribed certain dimenfions neceffary to the fize of the piece of bark to be applied, I have never obferved any general rule in that refpect, but have varied its fize according to the age of the patient or fize of his arm, or to the degree of inflammation and fuppuration I wifhed to take place.

If

If thefe trifling Obfervations find room in your refpectable Medical Mifcellany, I fhall continue to communicate to you a variety of foreign practice, not generally known in England; or, if known in terms, not frequently in application.

I am, &c.

*Great Russel Street, Bloomsbury,*

*Feb.* 16, 1801.

J. ROGERS.

